# Supplementary material for: A Tryptophan ‘Gate’ in the CRISPR-Cas3 Nuclease Controls ssDNA Entry into the Nuclease Site, That When Removed Results in Nuclease Hyperactivity
Source: Int J Mol Sci. 2021 Mar 11;22(6):2848. doi: 10.3390/ijms22062848 (PMC8001533; doi:10.3390/ijms22062848)
Supplement: Supplementary file 1 [file ijms-22-02848-s001.pdf]

SUPPORTING INFORMATION (SI)

A tryptophan 'gate' in the CRISPR-Cas3 nuclease controls ssDNA entry into the nuclease site, that when removed results in nuclease hyperactivity.

Liu He[1], Zoe Jelić Matošević[3], Damjan Mitić[2], Dora Markulin[2], Tom Killelea[1], Marija Matković[4], Branimir Bertoša[3], Ivana Ivančić-Baće[2*] and Edward L. Bolt[1*]

[1.] School of Life Sciences, University of Nottingham, United Kingdom.
[2.] Department of Biology, Faculty of Science, University of Zagreb, Croatia.
[3.] Department of Chemistry, Faculty of Science, University of Zagreb, Croatia.
[4.] Institute Ruđer Bošković, Zagreb, Croatia.
***** Correspondence: ed.bolt@nottingham.ac.uk or iibace@biol.pmf.hr

Contents of Supporting Information:

1. **Supplementary materials.** Strains and plasmids used in this study.
2. **Figure S1.** Fork DNA substrate.
3. **Figure S2.** CD monitoring of wild type Cas3 thermal denaturation.
4. **Figure S3.** EMSAs showing wild-type Cas3 and Cas3^{W406A} DNA-protein complexes.
5. **Figure S4.** CD monitoring of Cas3^{W406A} thermal denaturation.
6. **Figure S5.** Quantification of the distance between the centre of phenyl rings of Trp-230 and Trp-406 and the most prominent hydrophobic interactions made by Trp-406 during the molecular dynamics (MD) simulations.
7. **Figure S6.** Sequence alignment of *E. coli* Cas3 with the templates provided by SwissModel server.
8. **Figure S7.** Root mean square deviations (RMSD) of the protein backbone Cα atoms during the MD simulations.
9. **Table S1.** List of strains used in this study.
10. **Table S2.** List of oligonucleotides used in this study.
11. **Table S2.** Summary of molecular dynamics simulations conducted for different systems of *E. coli* Cas3 protein.
12. **Supplementary references.**

Supplementary materials

Strains and plasmids

The *E. coli* K-12 strains used in this study are described in the **Table S1**. Plasmids used were: pKOV (Link et al. 1997), pAH4 (*cas3* cloned into Bad-HisA using XhoI and EcoRI), pIIB39 (mutagenized pAH4 in W406A residue) using primers listed in Table 2 (oligos), pEB526 (Cas3 cloned in pUC19) (1). Plasmid pCas3 is described in (2).

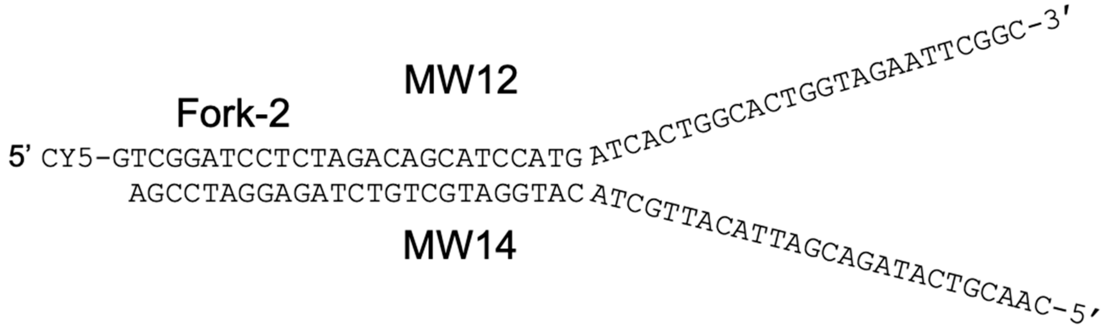

Figure S1. Fork DNA substrate. DNA oligos MW12 and MW14 were annealed to form a DNA fork substrate with 25 base pairs double-stranded region and extended two single-stranded 25 nt arms. A Cy5 fluorescent dye oligonucleotide labelling was incorporated at 5´-end of MW12 that has a maximal absorbance at 646 nm.

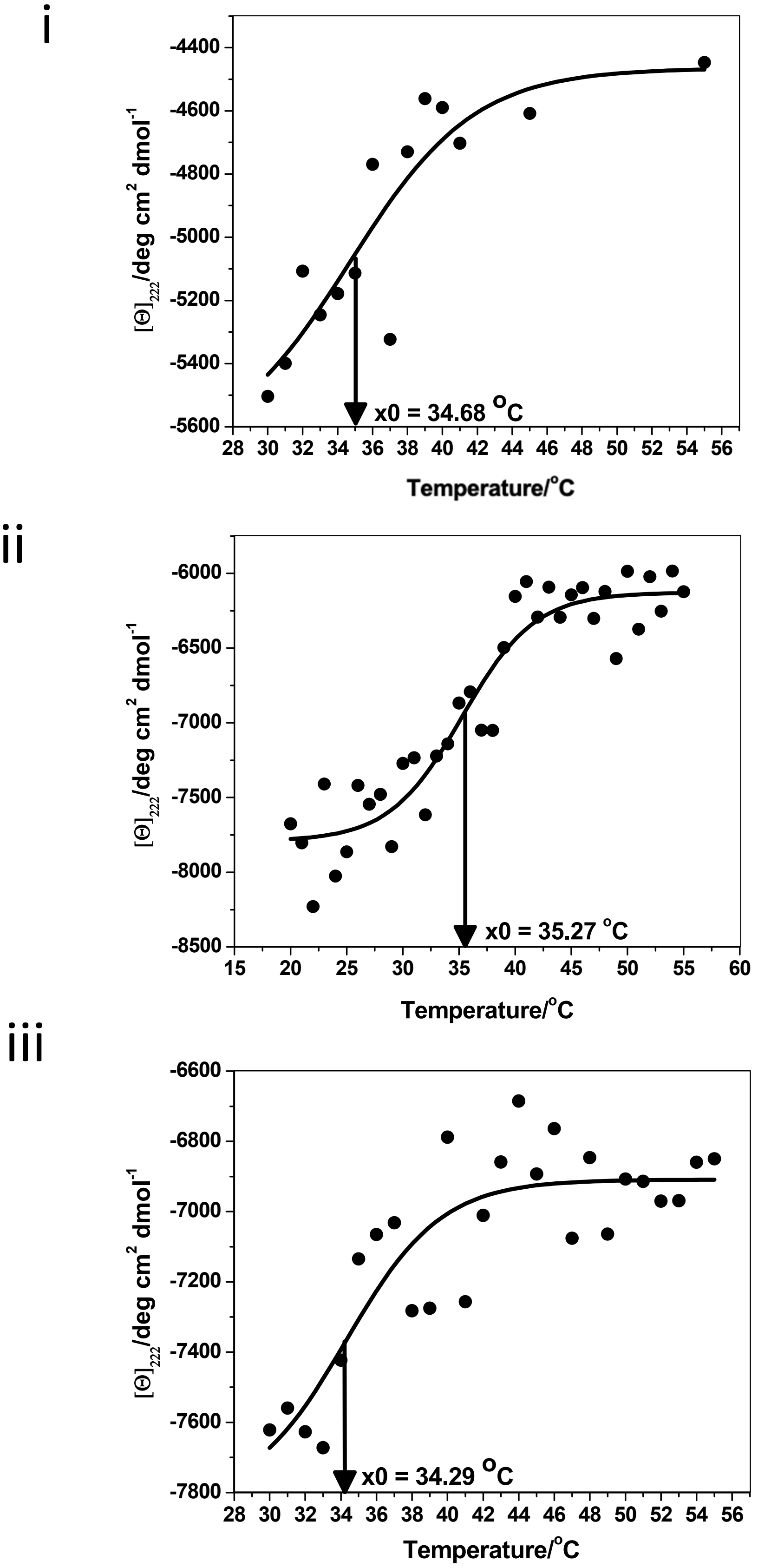

Figure S2. CD monitoring of wild type Cas3 thermal denaturation. (i). Replica1. CD monitoring of Cas3 thermal denaturation. Changes at 222 nm in temperature range 30-55 °C presented as mean residue ellipticity (MRE) *vs* Temperature ([Q]$_{222}$*vs*T): • experimental data — Boltzmann fit of experimental data; Boltzmann sigmoid value x0=34.68 °C; $|\Delta MRE_{exp}|=|MRE_{30}-MRE_{55}|=1056.56$

degcm^{2}dmol^{-1}, 19.2 %↓. **(ii).** Replica2. CD monitoring of Cas3 thermal denaturation. Changes at 222 nm in temperature range 20-55 ^oC presented as mean residue ellipticity (MRE) *vs* Temperature ($[Q]_{222}$*vs*T): • experimental data — Boltzmann fit of experimental data; Boltzmann sigmoid value x0=35.27 ^oC; l∆MREexpl=lMRE_{20}-MRE_{55}l= 1552.74 degcm^{2}dmol^{-1}, 20.2 %↓. **(iii).** Replica3. CD monitoring of Cas3 thermal denaturation. Changes at 222 nm in temperature range 30-55 ^oC presented as mean residue ellipticity (MRE) *vs* Temperature ($[Q]_{222}$*vs*T): • experimental data — Boltzmann fit of experimental data; Boltzmann sigmoid value x0=34.29 ^oC; l∆MREexpl=lMRE_{30}-MRE_{55}l= 771.81 degcm^{2}dmol^{-1}, 10.1 %↓.

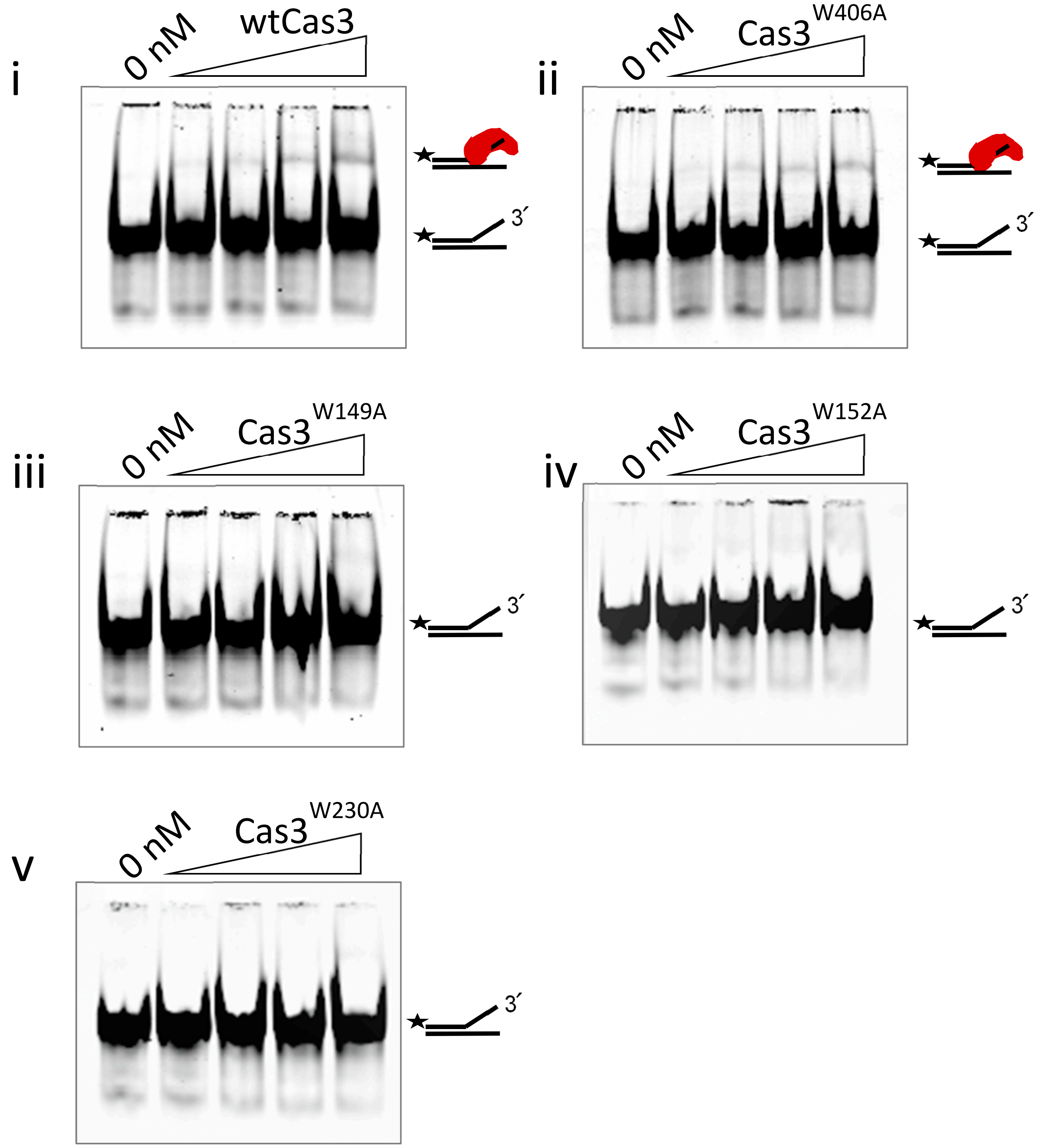

Figure S3. EMSAs showing wild-type Cas3 and Cas3^{W406A} DNA-protein complexes. EMSAs show that wild-type Cas3 and Cas3^{W406A} form stable DNA-protein complex (panels i and ii), but other mutant proteins do not (panels iii, iv and v). Increasing concentrations of Cas3 and mutant proteins (0, 0.4 0.8, 1.6 and 3.3 μM) were incubated with DNA fork (20 nM). Stable DNA-protein complex is indicated.

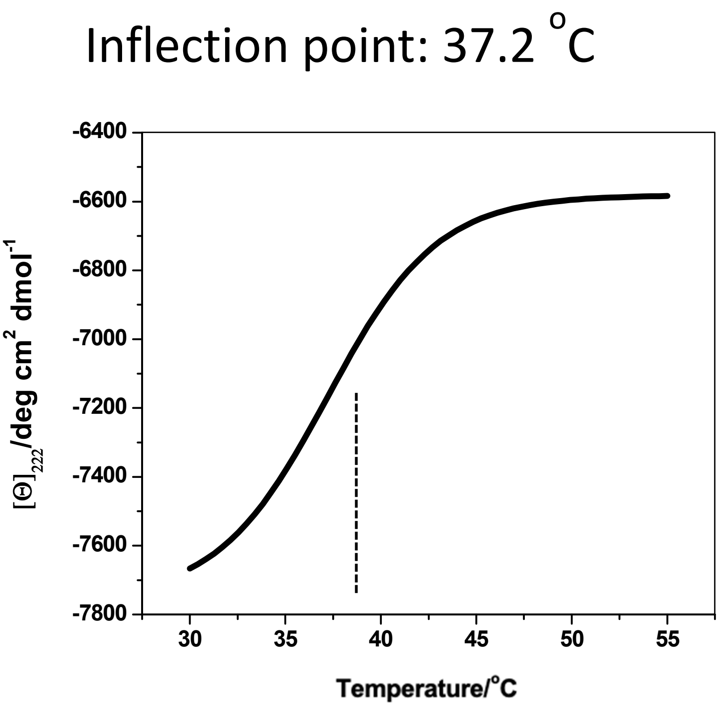

Figure S4. CD monitoring of Cas3^{W406A} thermal denaturation. Changes at 222 nm in temperature range 30-55 °C presented as mean residue ellipticity (MRE) vs Temperature ($[Q]_{222}$vsT): • experimental data — Boltzmann fit of experimental data; Boltzmann sigmoid value x0=37.20 °C; lΔMREexpl=lMRE_{30}-MRE_{55}l=1730.4 degcm^{2}dmol^{-1}, 14.0 %↓.

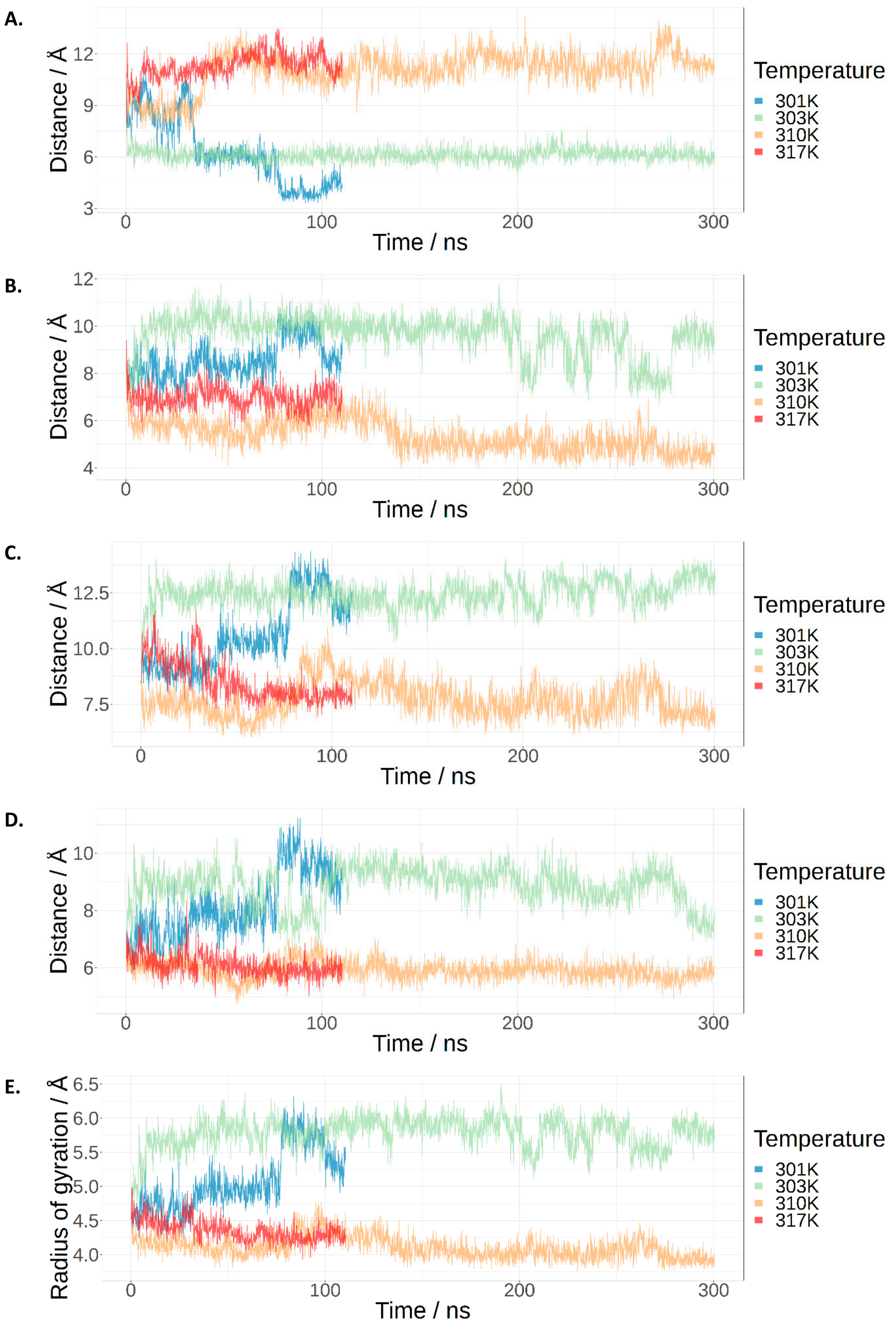

Figure S5. The distances between the centre of phenyl rings of Trp-230 and Trp-406 and the most prominent interactions that Trp-406 forms with the aliphatic part of sidechains of residues Val-415, Gln-426 and Arg-440 when entering a hydrophobic pocket at higher temperatures. The data shown

is from simulations of wild-type Cas3 protein complex with Mg^{2+} and ATP at 28 °C, 30 °C, 37 °C, and 44 °C. **A.** The distance between the sidechains of residues Trp-230 and Trp-406. **B.** The distance between the sidechains of residues Trp-406 and Val-415. **C.** The distance between the sidechain of Trp-406 and the aliphatic part of Gln-426 sidechain. **D.** The distance between the sidechain of Trp-406 and the aliphatic part of Arg-440 sidechain. **E.** The radius of gyration for the sidechains of Trp-406 and Val415 and the aliphatic part of Gln-425 and Arg-440 sidechains.

A.

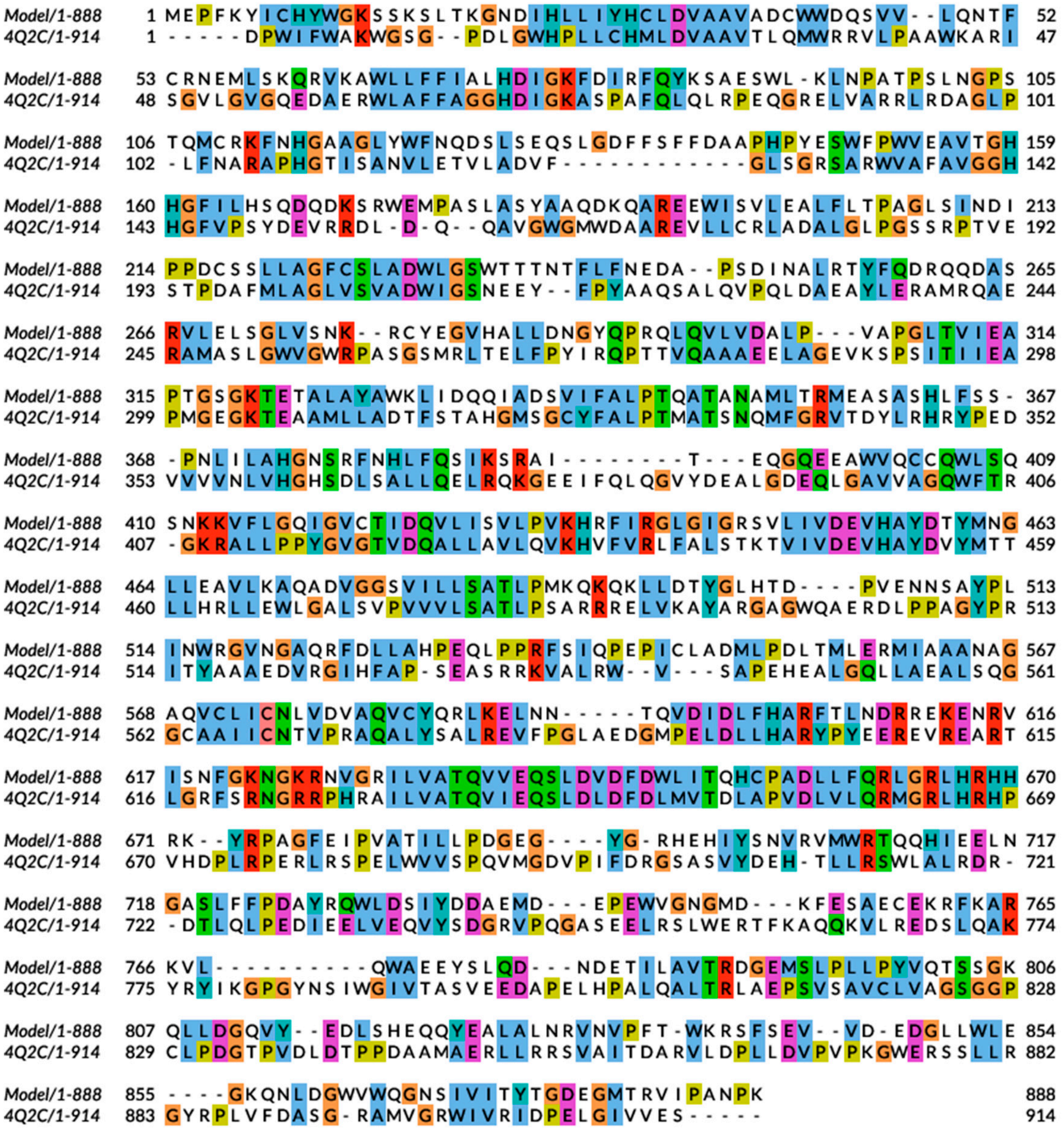

B.

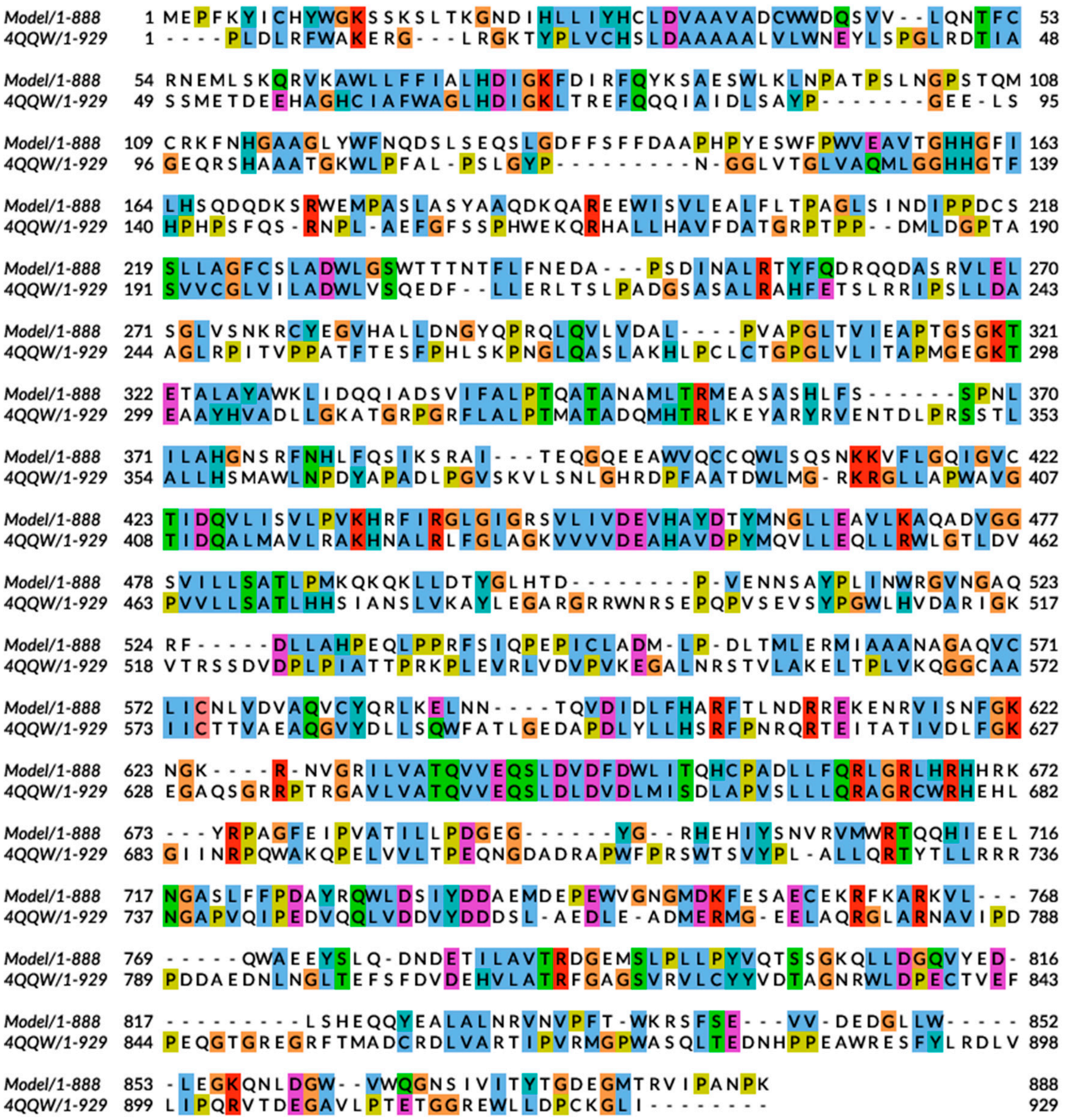

Figure S6. Sequence alignment of *E. coli* Cas3 with the templates provided by SwissModel server. A. Sequence alignment of *E. coli* Cas3 and *T. terrenum* Cas3 (PDB ID: 4Q2C). **B.** Sequence alignment of *E. coli* Cas3 and *T. fusca* Cas3 (PDB ID: 4QQW).

A.

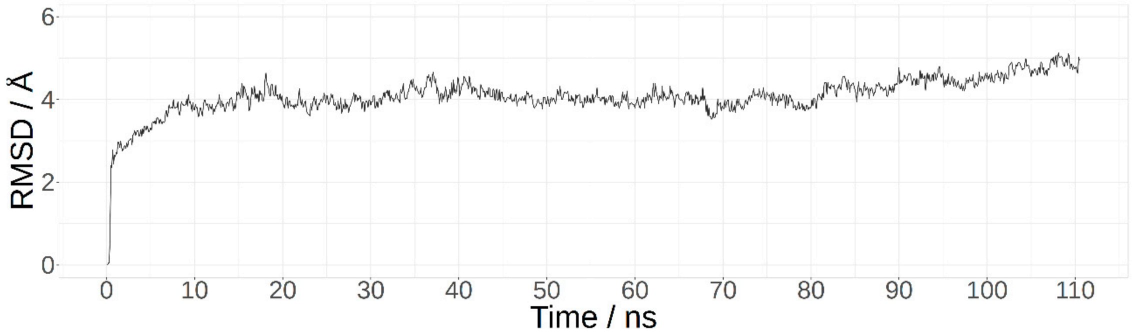
RMSD / Å
0 2 4 6
0 10 20 30 40 50 60 70 80 90 100 110
Time / ns

B.

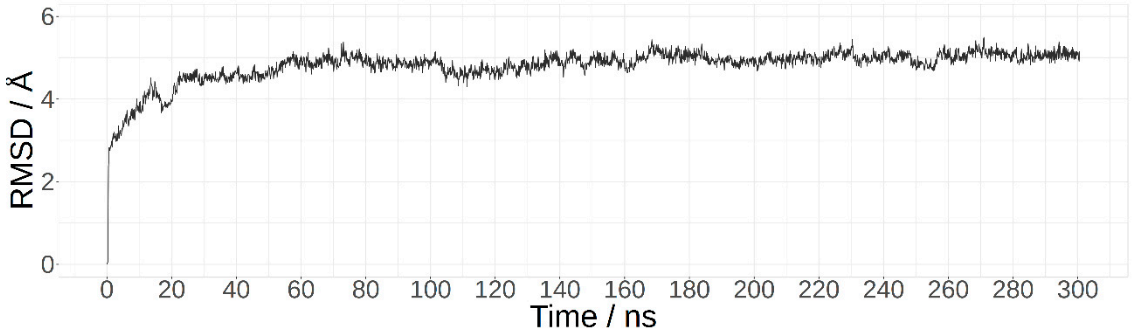
RMSD / Å
0 2 4 6
0 20 40 60 80 100 120 140 160 180 200 220 240 260 280 300
Time / ns

C.

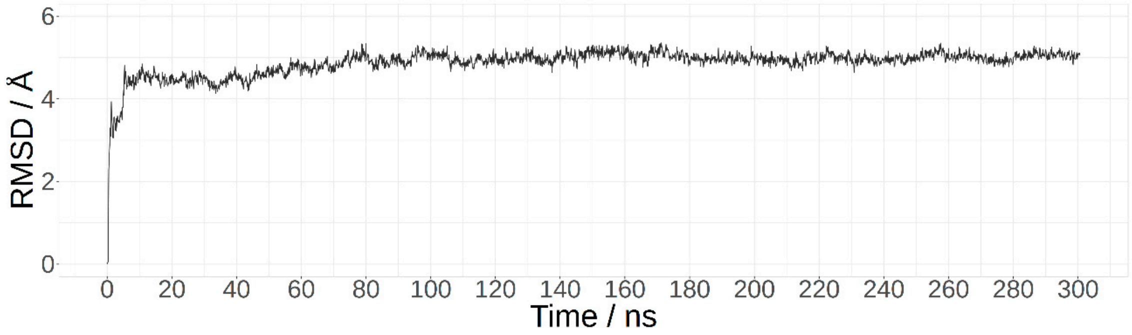
RMSD / Å
0 2 4 6
0 20 40 60 80 100 120 140 160 180 200 220 240 260 280 300
Time / ns

D.

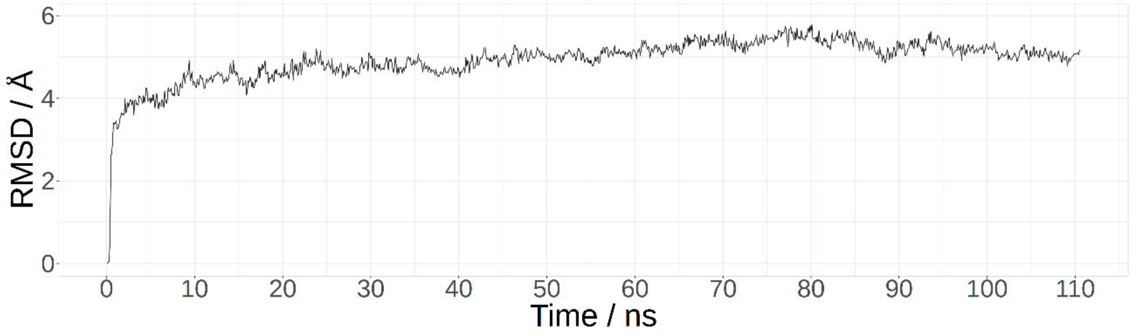
RMSD / Å
0 2 4 6
0 10 20 30 40 50 60 70 80 90 100 110
Time / ns

E.

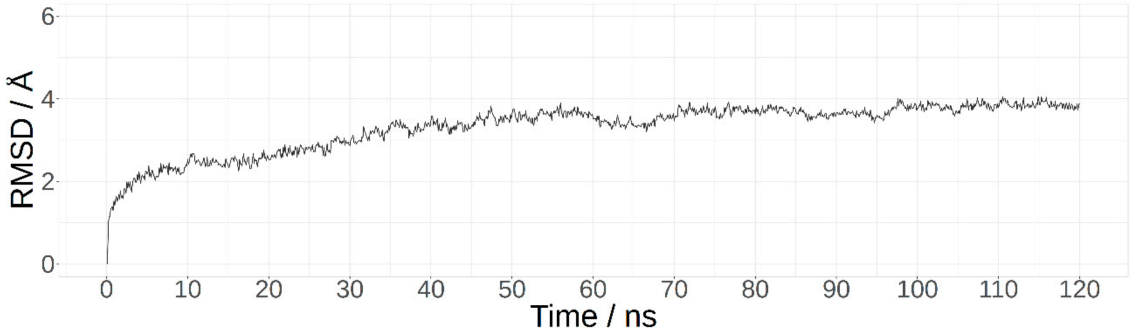
RMSD / Å
0 2 4 6
0 10 20 30 40 50 60 70 80 90 100 110 120
Time / ns

F.

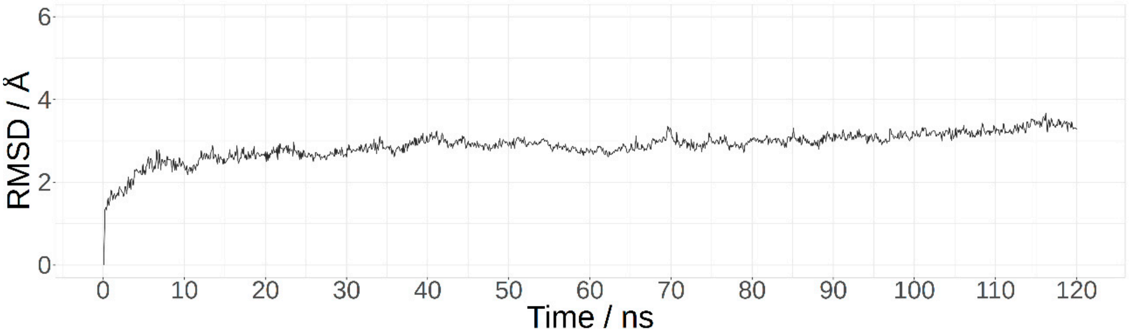
RMSD / Å
6
4
2
0
0 10 20 30 40 50 60 70 80 90 100 110 120
Time / ns

G.

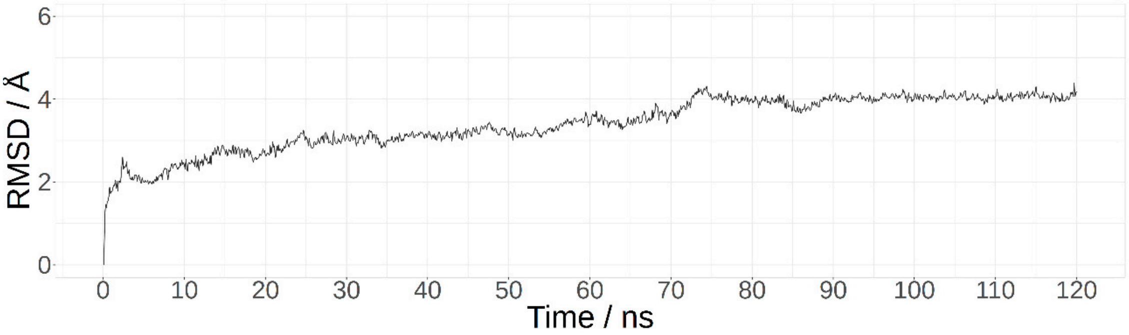
RMSD / Å
6
4
2
0
0 10 20 30 40 50 60 70 80 90 100 110 120
Time / ns

H.

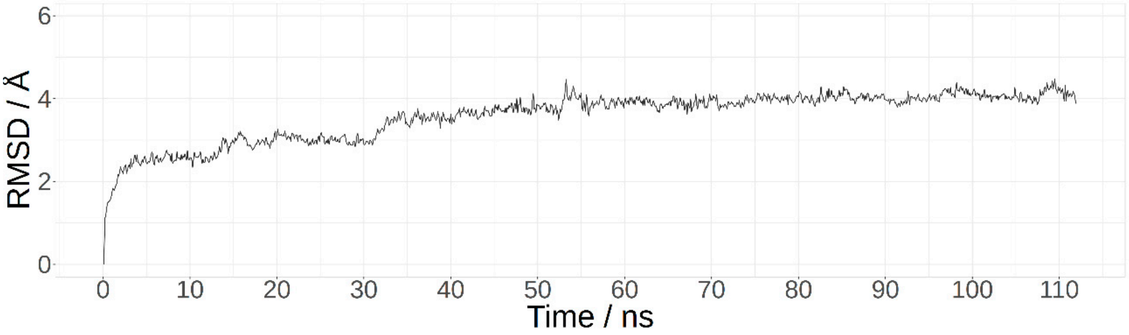
RMSD / Å
6
4
2
0
0 10 20 30 40 50 60 70 80 90 100 110
Time / ns

I.

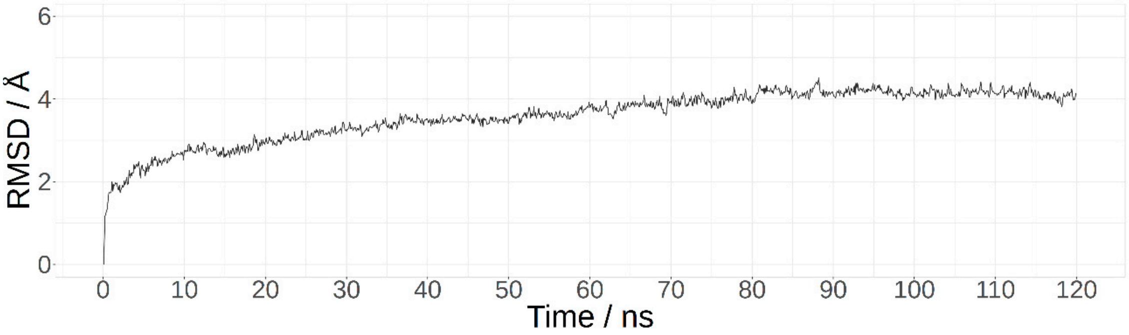
RMSD / Å
6
4
2
0
0 10 20 30 40 50 60 70 80 90 100 110 120
Time / ns

J.

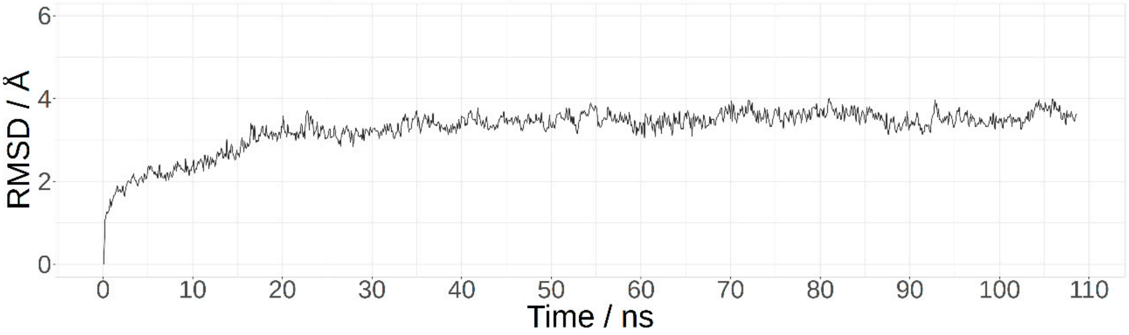
RMSD / Å
6
4
2
0
0 10 20 30 40 50 60 70 80 90 100 110
Time / ns

Figure S7. Root mean square deviations (RMSD) of the protein backbone Cα atoms. A. RMSD for simulation of wild-type protein in complex with Mg^{2+} and ATP at 301 K. **B.** RMSD for simulation of wild-type protein in complex with Mg^{2+} and ATP at 303 K. **C.** RMSD for simulation of wild-type protein in complex with Mg^{2+} and ATP at 310 K. **D.** RMSD for simulation of wild-type protein in complex with Mg^{2+} and ATP at 317 K. **E.** RMSD for simulation of wild-type protein in complex with Mg^{2+} and DNA at 303 K. **F.** RMSD for simulation of wild-type protein in complex with Mg^{2+} and DNA at 310 K. **G.** RMSD for simulation of W230A mutant in complex with Mg^{2+} and DNA at 303 K. **H.** RMSD for simulation of W230A mutant in complex with Mg^{2+} and DNA at 310 K. **I.** RMSD for simulation of W406A mutant in complex with Mg^{2+} and DNA at 303 K. **J.** RMSD for simulations of W406A mutant in complex with Mg^{2+} and DNA at 310 K.

Table S1. List of *E. coli* K-12 strains used in this study.

Bacterial strain	Relevant genotype	Source or reference
BW25113	F^- *rrnB* Δ*lacZ4748* (::*rrnB-3*) *hsdR514* Δ(*araBAD*)*567* Δ(*rhaBAD*)*568 rph-1* λ^-	(3)
	Bacterial strains related to BW25113	
BW39121	+ Δ*hns*::*kan*	(4)
IIB1040	+ λc + λT3 Δ*cas1*::*kan* Δ*hns*::*cat*	(5)
IIB1043	+λc + λT3 Δ*cas1*::FRT	(5)
IIB1309	+λc + λT3 Δ*cas1*::FRTΔ*hns*::*kan*	P1. BW39121 x IIB1043
IIB1342	+λc + λT3 Δ*cas1*::FRTΔ*hns*::*kan cas3W406A*	Gene replacement of *cas3* with allele *cas3*^{W406A} using pKOV – see main methods

Table S2. List of oligonucleotides used in this study.

Oligonucleotide Name	Sequence from 5' to 3'
Primers for amplyfing *ygcB* from genomic DNA	
ygcB_Forward	ATCGCTCGAGATAGAACCTTTTAAATATA
ygcB_Reverse	ATCGGCATGCTCGAATTCTTATTTGGGATTTGCAGGGA
Primers for DNA substrate preparation	
Cas3HD_Forward	GCCGCTCGAGGAACCTTTTAAATATATATGCCATT
W42A_Reverse	GCAACAGCAGCAACATCA
Primers for site-direct mutagenesis	
W149A_Forward	TTATGAGTCCgcgTTTCCATGGGTAGAGGC
W149A_Reverse	GGATGAGGAGCGGCATCA
W152A_Forward	CTGGTTTCCAgctGTAGAGGCCG
W152A_Reverse	GACTCATAAGGATGAGGAG
W230A_Forward	GCTTGCTGACgctTTAGGCTCCTGG
W230A_Reverse	GAGCAAAAACCTGCTAAC
W406A_Forward	GTGTTGTCAGgctTTGTCACAAAGCAATAAGAAAG
W406A_Reverse	TGAACCCACGCTTCTTCT
Primers for allele replacement	
FP-cas	TAAAAAAACAGGGAGGCTATTAGAATTAACCATGGGGGGTTC
RP-vector	ATCACTGAGATCATGTTGTAGCGCCCTTATTTGGGATTTGCAGGGATG
FP-promoter	CTATTGCTGGTTTANTCGGTACCCCAAGACATGTGTATATCACTG
RP-promoter	GAACCCCCCATGGTTAATTCTAATAGCCTCCCTGTTTTTTTAG
pKOV-F	GCAAATTCGACCCGGTCGTC
pKOV-R	GTTCCTGACCGATAACATCACAGA
Upcas3	CGATATTTATGAGCAGCATC
Downcas3	GATGTACATTGTGCACCTTC
Sequencing primer	
Cas3700	GGTTAGGCTCCTGGACTACAAC
Oligos for construct DNA fork substrate	
MW12	CY5-GTCGGATCCTCTAGACAGCTCCATGATCACTGGCACTGGTAGAATTCGGC
MW14	CAACGTCATAGACGATTACATTGCTACATGGAGCTGTCTAGAGGATCCGA
CRISPR RNA targeting sequence	AGGCCCGCACCGATCGCCCTTCCCAACAGTTG

Table S3. Summary of molecular dynamics simulations conducted for different systems of *E. coli* Cas3 protein.

	wild-type		W230A	W406A
	Mg^{2+}, ATP	Mg^{2+}, DNA	Mg^{2+}, DNA	Mg^{2+}, DNA
**Temperature / K**	**Time / ns**			
301	110	X	X	X
303	300	120	120	120
310	300	120	120	120
317	110	X	X	X

Supplementary References

1. Ivancic-Bace, I., Radovcic, M., Bockor, L., Howard, J.L. and Bolt, E.L. (2013) Cas3 stimulates runaway replication of a ColE1 plasmid in Escherichia coli and antagonises RNaseHI. *RNA Biol*, **10**, 770-778.
2. Mulepati, S. and Bailey, S. (2011) Structural and biochemical analysis of nuclease domain of clustered regularly interspaced short palindromic repeat (CRISPR)-associated protein 3 (Cas3). *J Biol Chem*, **286**, 31896-31903.
3. Datsenko, K.A. and Wanner, B.L. (2000) One-step inactivation of chromosomal genes in Escherichia coli K-12 using PCR products. *Proc Natl Acad Sci U S A*, **97**, 6640-6645.
4. Pougach, K., Semenova, E., Bogdanova, E., Datsenko, K.A., Djordjevic, M., Wanner, B.L. and Severinov, K. (2010) Transcription, processing and function of CRISPR cassettes in Escherichia coli. *Mol Microbiol*, **77**, 1367-1379.
5. Majsec, K., Bolt, E.L. and Ivancic-Bace, I. (2016) Cas3 is a limiting factor for CRISPR-Cas immunity in Escherichia coli cells lacking H-NS. *BMC Microbiol*, **16**, 28.
